# Human Q Fever on the Guiana Shield and Brazil: Recent Findings and Remaining Questions

**DOI:** 10.1007/s40475-021-00243-4

**Published:** 2021-06-01

**Authors:** Loïc Epelboin, Carole Eldin, Pauline Thill, Vincent Pommier de Santi, Philippe Abboud, Gaëlle Walter, Alessia Melzani, Paule Letertre-Gibert, Lucas Perez, Magalie Demar, Mathilde Boutrou, Jorlan Fernandes, Julman Rosiris Cermeño, Maria Mercedes Panizo, Stephen GS Vreden, Félix Djossou, Emmanuel Beillard, Jacobus H. de Waard, Elba Regina Sampaio de Lemos

**Affiliations:** 1 Infectious and Tropical Diseases Department, Centre Hospitalier de Cayenne Andrée Rosemon, Cayenne, French Guiana; 2 Centre d’Investigation Clinique (CIC INSERM 1424), Centre Hospitalier de Cayenne Andrée Rosemon, Cayenne, French Guiana; 3 Équipe EA 3593, Écosystèmes Amazoniens et Pathologie Tropicale (EPAT), Université de la Guyane, Cayenne, French Guiana; 4 Aix-Marseille Université, IRD, AP-HM, SSA, VITROME, Marseille, France; 5 IHU-Méditerranée Infection, Marseille, France; 6 Centre de référence pour la prise en charge des maladies vectorielles à tiques, Marseille, France; 7 Service Universitaire des Maladies Infectieuses et du Voyageur, Centre Hospitalier de Tourcoing, Tourcoing, France; 8 French Military Center for Epidemiology and Public Health, Marseille, France; 9 Laboratoire Hospitalo-Universitaire de Parasitologie-Mycologie, Centre hospitalier de Cayenne Andrée Rosemon, Cayenne, French Guiana; 10 Laboratório de Hantaviroses e Rickettsioses, Instituto Oswaldo Cruz, FIOCRUZ, Rio de Janeiro, RJ Brazil; 11 Departamento de Tuberculosis, Instituto de Biomedicina, Universidad Central de Venezuela, Caracas, Venezuela; 12 Mycology Department, National Institute of Hygiene Rafael Rangel, Caracas, Bolivarian Republic of Venezuela; 13 Internal Medicine and Infectious Diseases, Academic Hospital Paramaribo, Paramaribo, Suriname; 14 Laboratoire de Biologie Médicale, Institut Pasteur de la Guyane, Cayenne, French Guiana; 15 Departamento de Parasitología y Microbiología, Escuela de Ciencias de la Salud “Dr. Francisco Battistini Casalta” - Universidad de Oriente, Núcleo Bolívar, Venezuela

**Keywords:** French Guiana, Suriname, Guyana, Brazil, Venezuela, Guiana Shield, Latin America, Q fever, Zoonosis, *Coxiella burnetii*, Community-acquired pneumonia

## Abstract

**Purpose of Review:**

In this review, we report on the state of knowledge about human Q fever in Brazil and on the Guiana Shield, an Amazonian region located in northeastern South America. There is a contrast between French Guiana, where the incidence of this disease is the highest in the world, and other countries where this disease is practically non-existent.

**Recent Findings:**

Recent findings are essentially in French Guiana where a unique strain MST17 has been identified; it is probably more virulent than those usually found with a particularly marked pulmonary tropism, a mysterious animal reservoir, a geographical distribution that raises questions.

**Summary:**

Q fever is a bacterial zoonosis due to *Coxiella burnetii* that has been reported worldwide. On the Guiana Shield, a region mostly covered by Amazonian forest, which encompasses the Venezuelan State of Bolivar, Guyana, Suriname, French Guiana, and the Brazilian State of Amapá, the situation is very heterogeneous. While French Guiana is the region reporting the highest incidence of this disease in the world, with a single infecting clone (MST 117) and a unique epidemiological cycle, it has hardly ever been reported in other countries in the region. This absence of cases raises many questions and is probably due to massive under-diagnosis. Studies should estimate comprehensively the true burden of this disease in the region.

## Introduction

**Fig. 1:**
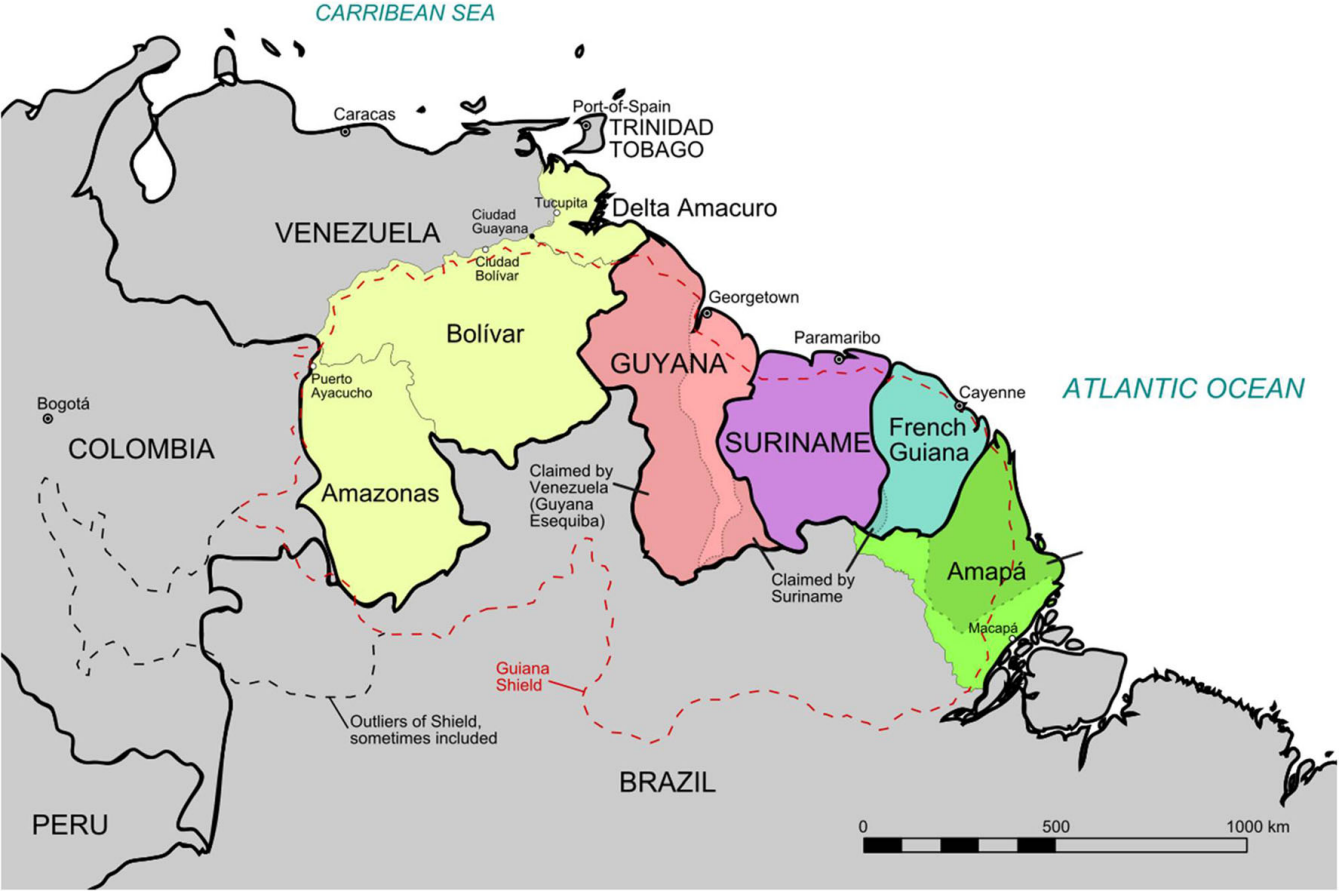
Map of the Guiana Shield. Adapted from Arnold Platon - own work, based on this map and the map from this article, CC BY-SA 3.0, https://commons.wikimedia.org/w/index.php?curid=18445142

Q fever has been a major focus of research in French Guiana (FG) for two decades. Although the veil is gradually lifting on many of its mysteries, its unique epidemiology, its clinical presentation, its temporo-spatial distribution, its risk factors, its animal reservoir, and its modes of transmission will still occupy clinicians and researchers for years to come. We here present an update of this singular zoonosis on the Guiana Shield—a region of northeast South America including Bolivar State in Venezuela, Guyana (previously British Guiana), Suriname (previously Dutch Guiana), French Guiana, and the State of Amapá in Brazil (Fig. 1). We also provided an update on the knowledge of Q fever in Brazil.

### General Information on Q Fever

Q fever is a zoonosis caused by the intracellular bacterium *Coxiella burnetii*, *Cb*, first described in Australia in 1935 [1]. The animal reservoir includes domestic mammals, mainly small ruminants (sheep, goats), more rarely cattle, pigs, dogs, cats, rabbits, and also many species of mammals, and wild birds. Infected mammals excrete *Cb* in urine, feces, milk, and birth products [1].

In humans, exposure most often results from inhalation of aerosols contaminated by amniotic fluid, animal placenta, or the release of *Cb* into the environment via excreta. Under certain conditions, the bacteria can be spread by wind, so the disease can occur in people without any contact with animals. Q fever is seen more commonly in men between the ages of 30 and 70 years. Risk factors include life in rural areas, consumption of raw milk or raw milk cheese, environmental or occupational exposure (breeders, farmers, and laboratory personnel), and contact with infected animals during gestation, newborn animals or during parturition [2].

Q fever is ubiquitous, absent only in New Zealand. Its observed incidence varies from country to country, depending on the frequency of breeding, doctors’ knowledge of the disease, the availability of microbiological diagnostic techniques, and the existence of dedicated epidemiological surveillance. Depending on the geographical area, endemic or epidemic situations are observed [1]. In endemic areas, cases occur, usually after risky activities (agriculture, slaughterhouse work, or rural tourism). This is the case in France, Spain, Greece, Israel, and the USA. The incidence of the disease is then linked to exposure to ruminants. Seasonality is observed, with two peaks of incidence in spring and early summer. Finally, large-scale epidemics can occur as in the Netherlands between 2007 and 2010, faced with the largest epidemic of Q fever ever observed, with more than 4000 cases declared, and more than 40,000 cases estimated [3].

In humans, acute Q fever usually presents with isolated fever, pneumonia, and/or acute hepatitis [2]. Chronic Q fever, more recently renamed as persistent localized, manifests in two main types: infectious endocarditis and vascular infection, although other impairments have been described [4–6].

### Q Fever in French Guiana, the Highest Incidence in the World

**Fig. 2:**
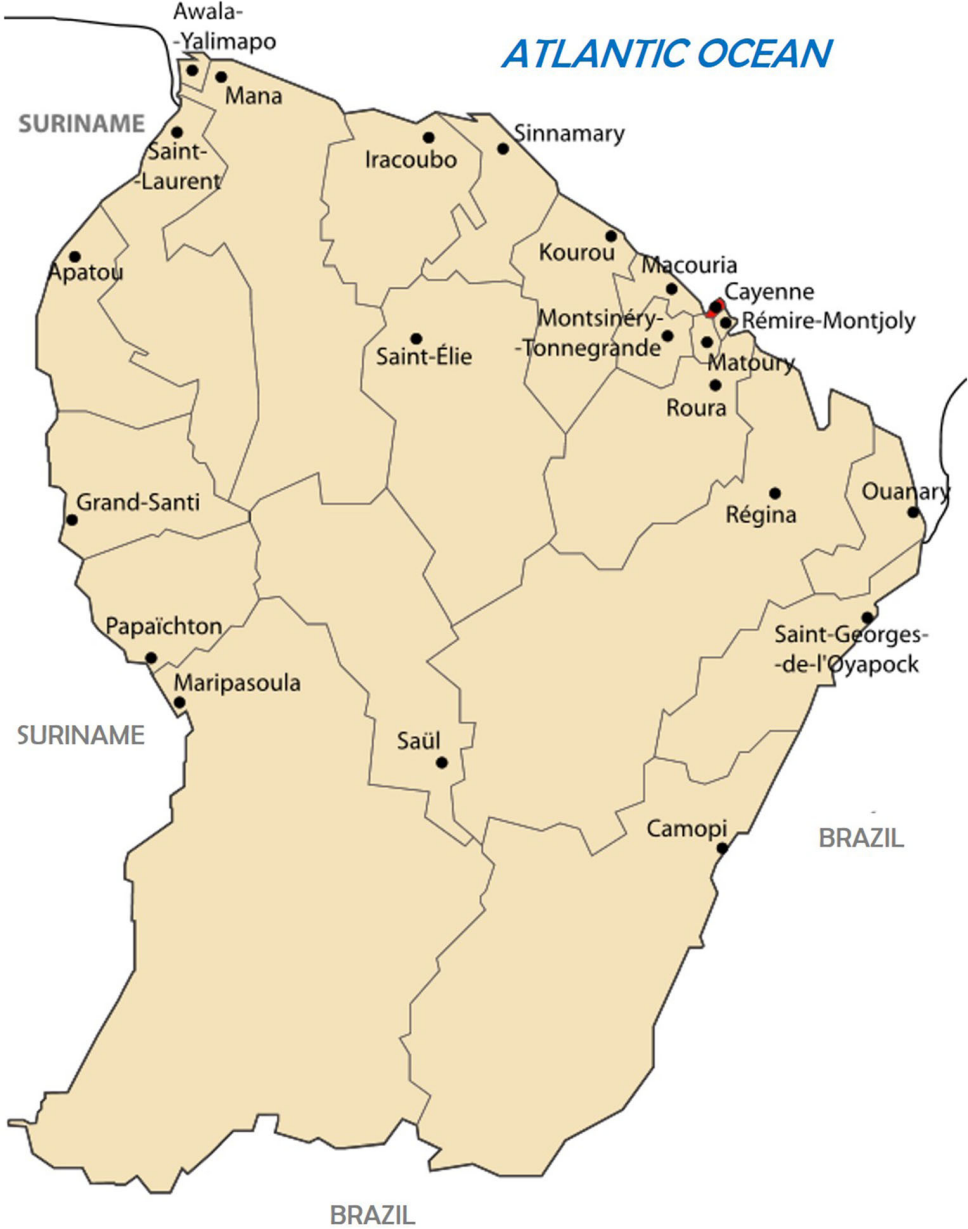
Map of French Guiana with the 22 municipalities; adapted fromKimdime69 - own work, CC BY 2.5, https://commons.wikimedia.org/w/index.php?curid=1896863

French Guiana is a French Overseas Territory of the Americas along with the islands of Martinique and Guadeloupe. The official population is around 275,000 inhabitants, it is multicultural and mainly concentrated on the coastline, half of which live and the greater Cayenne area including the municipalities of Cayenne, Rémire-Montjoly, and Matoury (Fig. 2). Although today Q fever in FG elicits many questions and publications, its exceptional characteristics were barely known until the mid-1990s. Identified by serology in cattle and humans in the 1950s, only a few sporadic human cases were described until recently [7, 8]. In 1996, three patients with Q fever were hospitalized in intensive care at the Cayenne Hospital Center for acute respiratory distress, one of whom died [9].

In order to assess whether these cases were linked to an increase in the incidence of the disease or to an improvement in its diagnosis, a sero-epidemiological investigation was then undertaken [9, 10], and showed a significant increase in the prevalence of positive serologies between 1992 and 1996: (1.9% in 1992, 9.1% in 1993, 8.6% in 1994, 4.8% in 1995, and 23.9% in 1996) [10].

In 2001, a large study undertook to measure the incidence and risk factors for Q fever, but also to identify its animal reservoir in FG [11]. A total of 132 cases of Q fever were diagnosed between July 1996 and October 2000, 70.5% of which were men, representing an average incidence rate of 37 cases per 100,000 inhabitants per year, which is higher than mainland France but close hyperendemic foci in the South of France.

In 2007, the Institut de Veille Sanitaire and the Institut Pasteur in FG published a retrospective serological study based on samples collected from 1950 to 2006, in order to assess the incidence of Q fever in FG [12]. A total of 1692 probable cases with positive serology (acute or persistent focused form) were identified. By contrast, during the 1950–1989 period: only 10 probable cases were identified. After a peak observed in 1997–1998, the number of cases decreased until 2002, then increased to reach its maximum in 2005 with 286 cases. The annual incidence rate was 9.3 cases p. 100,000 in 1996 and then 149.9 cases p. 100,000 in 2005. Over the 2003–2005 period, the incidence rate in FG was thus 160 to 500 times higher than in mainland France (0.32 to 0.58 cases p. 100,000/year).

A more recent incidence study carried out between 2007 and 2017 showed that from 2010 onwards, the incidence rate remained relatively stable—but very high—between 27 and 40 cases p. 100,000/year, thus confirming the categorization as a state of hyperendemicity [13•]. By way of comparison, the annual incidence rate p. 100,000 inhabitants is 0.02 for South Korea, 2.5 for France, 0.38 for Taiwan, and 2.8 for Australia [14].

### High Prevalence of Acute Q Fever Among Community-Acquired Pneumonias in Cayenne Area

One of the main particularities of Q fever in FG is the overrepresentation of pulmonary forms. A study between 2008 and 2011 showed that pneumonia represented 83% of Q fever cases in FG whereas it was 8 to 37% in mainland France [15]. *Cb* is the causative pathogen for less than 1% of hospitalized acute community lung disease (CAP) in the UK and continental Europe, 2.3% in North America, and 5.8% in Israel, a highly endemic region [16]. In FG, two consecutive studies at Cayenne hospital have shown that *Cb* was responsible for 24.4% (2004–2007) to 38.5% (2008 and 2012) of hospitalized CAP. The proportion of Q fever among CAP in FG seems therefore to be by far the highest in the world.

Consequently, *Cb* must be systematically suspected and treated in the event of any fever in FG or in a traveler returning from FG by empiric antibiotic therapy comprising an anti-*Cb* agent (doxycycline) and an anti-pneumococcal agent—usually a β-lactam. The Cayenne study published in 2012 proposed a score differentiating pneumonia caused by *Cb* from those caused by another organism [17]. Thus, the presence of headache, male gender, age between 30 and 60 years, and a high C-Reactive Protein level (> 185 mg/L) associated with normal leukocytes (<10 G/L) were strongly in favor of Q fever.

### Incidence of Persistent Focalized Infection by *Coxiella burnetii* (Formerly Chronic Q Fever)

Few data are available regarding persistent focused Q fever in FG. A study compared the epidemiological profile of Q fever in FG with that of Marseille between January 2008 and December 2011: eight patients presented with endocarditis but none with vascular infection, vs. 31 in Marseille during the same period. This led the authors to conclude that cardiovascular involvement was significantly rarer in FG than in Marseille with an annual cumulative incidence during these 4 years for Q fever–associated endocarditis in the Cayenne area (1.29 cases p. 100,000 inhabitants) not significantly different than the incidence in the Marseille area (0.34 cases p. 100,000 inhabitants) (p = 0.45) [15]. A medical thesis studying 52 cases of endocarditis treated at Cayenne hospital between 2007 and 2013 found that 39 (75%) were microbiologically documented, of which 3 (5.8%) incriminated *Cb* (Le Labourier, 2015). A retrospective study of the incidence of persistent focused Q fever in FG between January 2008 and December 2016 was also conducted: 51 confirmed or probable cases were identified [18]. Depending on the numerator chosen (confirmed vs. probable) and the denominator chosen (population of FG vs. that of Cayenne municipality), the incidence varied from 0.47 to 5.21 cases p. 100,000 inhabitants per year, while the annual incidence of *Cb* endocarditis in France at the same time was estimated at 0.1 p. 100,000 inhabitants/year.

### Unique Geographic and Climatic Features

In the first study published in 1998, the majority of cases of Q fever concerned persons living in the urban municipality of Cayenne where *Cb* seroprevalence was higher than in rural areas (21/161 (13.0%) vs. 4/114 (3.5%); p < 0.01), a very unusual phenomenon for a generally rural zoonosis [9]. In the study published in 2001, only 5.3% of cases (7/132) were located outside the Cayenne and surroundings (i.e., cities of Cayenne, Matoury, and Rémire-Montjoly municipalities) [11]. No case of Q fever has been recorded in the hospitals of Kourou and Saint Laurent du Maroni, located respectively 50 and 250 km from Cayenne. The cases identified in Cayenne and its surroundings were widely dispersed, which does not really support the hypothesis described in other urban epidemics of the spread of bacteria by the wind from a single source, suggesting a reservoir probably different from those usually described, namely farm animals, in which serologies have been found to be of little contribution. A second study of 106 *Cb* vs. 169 other CAP hospitalized between 2008 and 2012 showed that 85.3% of patients lived in Cayenne and surroundings [19], more particularly in Rémire-Montjoly for the Q fever group compared to the control group (29.4% vs. 18.3% p = 0.04) and significantly fewer patients with Q fever living outside the Cayenne area (5.9% vs. 15.2%, p = 0.02). The study by Thill et al. observed a similar result for the island of Cayenne where 86% of the 700 cases identified between 2007 and 2017 lived [13•]. Residing in Cayenne and surroundings was a significant risk factor for infection (p < 0.01; OR = 6.05; 95% CI = 4.88–7.55). However, a recent seroprevalence study conducted by the Institute Pasteur in FG on 2697 individuals drawn by lot in the 22 communes of FG described a different phenomenon [20••]. Thus, 9.6% [8.2–11.0%] of the Guianese population had phase II IgG. Although the municipalities of Cayenne and surroundings had particularly high seroprevalence, some municipalities such as Maripasoula had a seroprevalence > 10.0%, although the clinical cases reported in persons living in this municipality of nearly 10,000 inhabitants are rare. Finally, the temporal distribution of cases is also unusual in FG, since we find a correlation with rainfall and the alternation between the dry season and the rainy season. Thus, Gardon et al., in 2001, observed peaks of incidence once to twice a year (in July, at the start of the long dry season, and in March, at the start of the short dry season), and a decrease of transmission between the rainy period between December and February [11]. The increased incidence at the start of the dry season could be explained by the aerosolization in the dust of bacteria present in the environment.

### Unusual Exposure Factors

The study by Gardon et al., in 2001, studied the risk factors for Q fever and its results were surprising: occupation in the field of buildings and public works, seeing bats or non-flying wild mammals near home, and living near the forest were significant risk factors [11]. In 2007, a study of the exposure factors of 24 patients hospitalized for Q fever showed that nearly 50.0% of them had been in contact with bat or poultry droppings; the remainder had been exposed to aerosolization of soil during groundworks or to a slaughterhouse [21]. It was thus concluded that the aerosolization of environmental products could lead to inhalation of bacteria, and therefore explain the predominance of pneumonia patterns encountered in acute Q fever in FG.

Two successive studies carried out at Cayenne General Hospital on the risk factors of CAP to *Cb* identified identical factors: being between 30 and 60 years old, male gender, and being from mainland France [14, 17]. In the second study, gardening was an additional risk factor. The incidence study carried out between 2007 and 2017 compared the demographic characteristics of the 700 cases of Q fever to those of the general population and identified the same risk factors: being between 30 and 60 years old, being a male, living in the greater Cayenne area, and originating from mainland France [13•].

From December 2012 to June 2013, a Q fever epidemic occurred on the Tiger Mountain military site [22]. Among the 54 occupants of the premises, 11 developed the disease (attack rate 11/54 = 20%) and 50% (8/16) of the households were affected. The two main exposure factors found were cleaning (with a broom) and carrying a sloth in one’s arms. The conclusion was that the probable mode of contamination was inhalation of dust contaminated with *Cb* by resuspension during household activity. Statistical and microbiological analyses suggested a role for the sloth and/or its ticks in this epidemic.

In August 2014, 5 soldiers out of 12 of the French Navy developed an acute Q fever after a stay in the forest on the banks of the Comté River, 25 km from Cayenne [23]. The main risk factor identified was having used a brush cutter. No asymptomatic seroconversion was observed in these two studies.

### An Exceptional Case of Human-to-Human Transmission

Although human-to-human transmission of Q fever is anecdotal, it has been documented in the context of birth. Indeed, case of *Cb* pneumonia was diagnosed in an obstetrician 7 days after delivering the infant from an infected woman [24]. In addition, nosocomial transmission between two pregnant women sharing the same room has been reported [25]. In this case, the most likely route of infection was aerosolized infectious particles excreted through the vagina. Another case was supported by the FG team (Epelboin, unpublished data). A woman and her companion living in FG left to join her brother, living in a large Canadian city, for a vacation in New York, in the middle of winter. As soon as he arrived, the companion developed a feverish cough, which was labeled Q fever on his return to FG. The woman and her brother spend a week locked in the hotel room with the coughing patient, and then each returned to their respective countries. The woman and her brother who lived in Canada both developed Q fever upon returning from New York. Although nothing can be concluded from the mode of contamination of the woman, the Canadian man was not exposed to any particular risk factor except having shared a New York hotel room confined for 1 week with a patient infected with *Cb*.

### A Mysterious Animal Reservoir

From the first studies on Q fever in FG, it appeared that *Cb* was not found in the classic reservoirs, mainly small domestic ruminants, goats, and sheep. Thus, during the study carried out at the end of the 1990s, serologies were carried out on sera from bovines, sheep, and goats. Among the animal herds tested, 6 cattle (1.7%) had anti-*Cb* antibodies at low levels, but no sheep or goats did [9]. During the study by Gardon et al., several exposure factors, such as the proximity of the forest to human dwellings, the fact of seeing small mammals and bats from the house, had suggested a reservoir in the wild fauna [11]. Several studies in search of an animal reservoir have been successively carried out in domestic and wild fauna, rodents, marsupials, birds, bats, cattle, dogs, and cats with serological surveys and search for carriage by PCR, none of which have been conclusive [11]. During the 2013 investigation at the Montagne du Tigre site, a three-toed sloth (*Bradypus tridactylus*) was found dead on the side of the road. The samples taken from it were positive in the feces and spleen by PCR, as well as its ticks [26]. It was therefore the first positive sample from an animal in FG for Q fever. This result corroborated the investigation carried out in humans from the Montagne du Tigre which found as an exposure factor having carried this animal in one’s arms [22]. However, *Cb*’s research on three swabs of 3 other three-toed sloths, a male and his 55 ticks, and a female, her calf, and her 30 ticks were negative during the investigation at the same place, as well as several individuals of an association for the protection of sloths, which are against pinning the endemic Q fever in FG solely on sloths. More recently, the PCR analysis of capybara droppings (*Hydrochoerus hydrochaeris*) was positive for *Cb*, this analysis was performed in the investigation of the cluster that occurred among the sailors of the French Navy at the Comté river [23]. Other species have also been found anecdotally positive for *Cb*: the collared peccary (*Tayassu pecari*) and the white-lipped peccary (*Pecari tajacu*) (Amazonian wild boar species) [27]. On the other hand, while part of the Guianese population is convinced that Q fever is transmitted by bats, no study has shown it.

### MST 17: the Only Strain and a More Virulent Strain Only Found in French Guiana

Early on, the possibility of the greater virulence of the Guianese strain of Q fever was raised. One clinical argument was the greater frequency of lung involvement and fever and the lower frequency of asymptomatic forms than in mainland France (97% versus 81% in Marseille, p < 0.0001) [15]. In addition, the study published in 2012 comparing CAPs linked to *Cb* to other causes showed generally “noisier” clinical pictures in Q fever than in other causes of acute pneumonia [17]. A study comparing Guianese and Marseille acute Q fevers showed a stronger and more prolonged immune response in French Guianese patients after acute Q fever [15]. Nevertheless, a medical thesis of medicine showed that Q fever was barely incriminated in severe CAP (Courcol, unpublished data). They showed that between 2011 and 2015 among 82 patients admitted for severe CAP in Cayenne Hospital’s intensive care unit, only 2 (2.2%) were linked to *Cb*. However, very few of them had a systematical serodiagnosis test at day 21, which is the gold standard for Q fever diagnosis

Finally, in 2013, the Guianese strain was successfully isolated from early samples from 5 patients of FG [28]. All samples contained the same genotypically characterized clone: MST 17. This genotype was also found in 2013 in 3 patients of the Tiger Mountain epidemic, as well as in sloths and their ticks [26]. Since then, this genotype is the only one to have been identified in patients from FG and has only been found in patients who live or have lived in FG according to the national reference center [14].

More recently, the MST 17 (*Cb*175) strain has been sequenced and revealed a unique feature: a 6105 base pair deletion in the hlyCABD operon of the type 1 secretion system (T1SS). This deletion was detected only in the 8 other MST 17 strains but in none of the 298 strains in the CNR database [29]. This deletion is thought to be linked to its exceptional pathogenicity. Finally, the MST 17 strain is resistant to macrolides (erythromycin and azithromycin) in vitro but remains sensitive to doxycycline, minocycline, levofloxacin, sulfamethoxazole/trimethoprim, and tigecycline [30]. A study performed in Cayenne showed that people treated for acute Q fever with macrolides were more at risk of developing persistent Q fever than those treated with doxycycline [31•].

A study recently compared three different *Cb* strains in vivo: the FG strain (*Cb*175), a German strain (Z3055, clone of the Dutch strain), and the reference strain Nine Mile (RSA 493) [32]. The FG strain showed greater mice mortality rate (100% at 4 weeks) than the German strain (75% at 4 weeks) or the Nine Mile strain. In addition, the French Guianese and German strains induced a significantly higher serological response at 2 and 4 weeks after infection (p < 0.05) than the Nine Mile strain, confirming that the FG strain was the most virulent.

### Q Fever Elsewhere on the Guiana Shield

Reports about *Cb* infection on the Guiana Shield are very scarce. According to our knowledge, no case of Q fever has been published from the Brazilian state of Amapá, Suriname, Guyana, and the Venezuelan State of Bolivar.

Nevertheless, one study was found from Amapá. The detection of anti-*Cb* antibodies by indirect immunofluorescence was performed in sera referred to the surveillance network of arboviruses of patients residing in the municipality of Oiapoque the city located on the border river separating Brazil from FG, from September 2014 to May 2015 [33]. Among 145 samples, 71 were analyzed and one was found positive in a woman with arthralgia, fever, myalgia, and headache, whose sample was obtained on the fifth day of illness. Furthermore, cases of acute pneumonia due to *C. burnetii* have been reported in Bolívar State of Venezuela, but so far not published (Rosiris Cermeño & De Waard, unpublished data)

The near absence of cases identified in the region may have several explanations. If we assume that the main clinical presentation in these countries very close geographically to FG is CAP, then we can imagine that the poor notoriously microbiological documentation of lower respiratory tract infections in general could make this diagnosis go unnoticed. As the course of these CAP is rarely severe, that is to say that they rarely lead to an admission in an intensive care unit (ICU) and are rarely the cause of death, even in the absence of appropriate antibiotic treatment, we can assume they are mistaken for slowly favorable *Streptococcus pneumoniae* CAP.

The microbiological diagnosis of Q fever requires immunofluorescence serodiagnosis techniques which are not readily available, especially in countries with lower resources, unlike FG which has resources equivalent to those of European countries.

Moreover, the presumed rarity of *Cb* endocarditis, even in FG where the acute forms are frequent, may explain why this diagnosis is rarely mentioned. This regional discrepancy warrants seroprevalence studies in the countries of the Guiana Shield, and the systematic search for Cb in the CAP, in order to test the hypothesis that Q fever is limited to the borders of French Guiana, or only reflects its greater diagnostic capacity and awareness, as had been assumed a few years ago [34].

### Q Fever in Latin America Outside the Guiana Shield

An exhaustive literature review published in 2016 had showed that publications on human Q fever in Latin America were rare [34]. Seven countries had never reported a case (Belize, Costa Rica, Guatemala, Guyana, Honduras, Paraguay, Suriname), three not since 1990 (Bolivia, Panama, Venezuela), while only one or two publications are available for seven countries since 1990 (Argentina, Chile, Ecuador, El Salvador, Peru, Trinidad, Uruguay). On the other hand, Colombia, Mexico, and especially Brazil had carried out several studies on seroprevalence in exposed populations or on prevalence within series of CAP. There are no publications on human Q fever in the Amazon region except in FG and Ecuador [35]. Finally, recently, the French National Reference Center described genotypes of Q fever according to the zone of presumed infection: no cases were reported in patients from South America, except for FG [14, 28].

It is of note that some CAP have been diagnosed in Venezuela but were not published (Rosiris Cermeño & De Waard, unpublished data), and a case had been reported in Spain in a traveler returning from Venezuela and the Dominican Republic [36].

### The Particular Case of Brazil

Taking Brazil as an example due to its continental proportion, despite the occurrence of confirmed cases of Q fever, the overall frequency can only be estimated from published cases but its real incidence is completely unknown, since this zoonosis can be mistaken with several infectious diseases that are frequently not recognized by most Brazilian health professionals. Although Q fever remains poorly understood and reported in Brazil, since the publication about the first case confirmed by PCR in a patient with fever of unknown origin and thrombocytosis in Rio de Janeiro state [37], the presence of *Cb* has been demonstrated in patients with influenza-like disease, associated with pneumonia. In a study conducted in 2016 in the municipality of Itaboraí, Rio de Janeiro state, blood samples from nine patients were *Cb*-PCR positive (3.3%) out of 272 patients admitted to the public hospital with suspected dengue infection [38]. Two years later, still in the Rio de Janeiro state, Lemos et al. described a cluster of Q fever among cadets with history of camping during military survival training where they participated in the slaughtering animals for their own consumption. Seroconversion was identified in all patients and *Cb* DNA was detected in bronchoalveolar lavage from one of the five cadets who had pneumonia with acute respiratory distress syndrome [39•, 40]. Previous prevalence studies in specific populations were carried out using immunofluorescence assays that showed a prevalence of 3.2% of *Cb* antibodies (4/125) in human immunodeficiency virus–positive patients and 9.3% (28/300) in individuals who injected drugs [41, 42]. In relation to animals, *Cb* infections have been reported not only in domestic and production animals, but also wild mammals, showing that this infection may be more prevalent than previously thought [43–47]. In a survey conducted in 2014 in the municipality of Itaboraí, Rio de Janeiro, *Cb* infection was detected in dogs, goats, and sheep [43, 44]. In addition, a study conducted in Alagoas state, Northeast Brazil, evaluated the *Cb* infection in 312 goats collected from a dairy flock with history of reproductive disorders. A total of 172 serum samples were ELISA seroreactive and two of 23 placental samples analyzed were positive by PCR [45]. Regarding wild animals, studies conducted in the Atlantic Forest in three Brazilian states (Bahia, Rio de Janeiro, and Santa Catarina) demonstrated *Cb* infection by molecular tests (PCR) in wild rodents 4.6% (6/131) and bats 3.4% (4/119) [46, 47]. Recent studies have also demonstrated a neglected aspect in *Cb*’s chain of transmission to humans—the report of this highly heat-resistant zoonotic pathogen in raw milk—, which could imply a potential threat to consumers [48]. Based on a random and representative baseline prevalence of fresh cheese made from raw milk from bovines, *Cb* DNA was detected in five samples (9.43% - 5/53). Authors then estimated that 1.62 tons/day of ready-to-eat cheese made from raw milk from a total of 16.2 tons produced daily in the study region were possibly contaminated with *Cb*, a neglected food-borne pathogen in Brazil, and probably in other Latin American countries [49].

### Q Fever’s Epidemiologic Silence in the Tropics at Large

Q fever is not commonly considered a tropical disease and much of the literature on Q fever originates from temperate countries and there is very little in much of the tropical world. For example, whether cases from Africa are scarce, there are some signals that let us think that the phenomenon is wider in the continent than usually thought. Thus, Eldin et al. wrote in a large literature review on Q fever that the infection is probably widespread in that continent, because several cases of Q fever have been reported in nine African countries in 1955 and several seroprevalence studies then revealed high seropositivity rates in several countries, especially those with high densities of ruminants [1, 50–53]. Since there is a great overlap between the geography of health expenditure per capita and the temperate/tropic divide, it is reasonable to suspect that absence of evidence for Q fever in the tropics is not evidence of its absence, but rather that laboratory capacity and well-funded research and epidemiological surveillance systems may explain much of the difference. Hence, the observations from the Guiana shield on this tropical pathogen could be a natural experiment—all countries are tropical, one is much richer—and Q fever is only found in there, suggesting that the epidemiology silence around Q fever in the tropics owns more to laboratory capacity than to tropical ecosystems.

## Conclusion

While FG has the highest incidence of Q fever in the world, countries and regions from the remaining Guiana Shield report no cases of this disease. This phenomenon is likely due to several factors, among which the absence of awareness and the low resources of the health systems probably play a major role. On the other hand, FG’s Q fever has specific characteristics, the explanation for which remains somewhat mysterious: the incidence is the highest in the world, its reservoir is still mysterious, and the FG strain MST17 could explain the essentially respiratory tropism and its increased virulence. Further studies are needed on the Guiana Shield and in the Amazon region to check if there is a specific epidemiology in FG, or if there is wide misdiagnosis in the surrounding countries. More generally, the alignment of epidemiological discrepancies regarding Q fever observed in a small ecologically homogeneous region with differences in health expenditure suggests that at a global scale the discrepancies between temperate/high resource countries and tropical low- and middle-income countries may have a similar explanation.

## Data Availability

Not applicable.
